# Supplementation with IL-6 and Muscle Cell Culture Conditioned Media Enhances Myogenic Differentiation of Adipose Tissue-Derived Stem Cells through STAT3 Activation

**DOI:** 10.3390/ijms19061557

**Published:** 2018-05-24

**Authors:** Eunhui Seo, Hwansu Kang, Oh-Kyung Lim, Hee-Sook Jun

**Affiliations:** 1 College of Pharmacy and Gachon Institute of Pharmaceutical Science, Gachon University, Incheon 21936, Korea; eunhuiseo@gachon.ac.kr (E.S.); hwansu83@naver.com (H.K.); 2 Lee Gil Ya Cancer and Diabetes Institute, Gachon University, Incheon 21999, Korea; 3 Department of Rehabilitation Medicine, Gil Hospital, Incheon 21565, Korea; phmed@gilhospital.com; 4 Gachon Medical Research Institute, Gil Hospital, Incheon 21565, Korea

**Keywords:** ADSCs, myogenic differentiation, IL-6, muscle cell conditioned media, STAT3

## Abstract

Mature skeletal muscle cells cannot be expanded in culture systems. Therefore, it is difficult to construct an in vitro model for muscle diseases. To establish an efficient protocol for myogenic differentiation of human adipose tissue-derived stem cells (hADSCs), we investigated whether addition of IL-6 and/or myocyte-conditioned media (CM) to conventional differentiation media can shorten the differentiation period. hADSCs were differentiated to myocytes using the conventional protocol or modified with the addition of 25 pg/mL IL-6 and/or C2C12 CM (25% *v*/*v*). The expression of MyoD and myogenine mRNA was significantly higher at 5–6 days after differentiation using the modified protocol than with the conventional protocol. mRNA and protein expression of myosin heavy chain, a marker of myotubes, was significantly upregulated at 28 and 42 days of differentiation using the modified protocol, and the level achieved after a 4-week differentiation period was similar to that achieved at 6 weeks using the conventional protocol. The expression of p-STAT3 was significantly increased when the modified protocol was used. Similarly, addition of colivelin, a STAT3 activator, instead of IL-6 and C2C12 CM, promoted the myogenic differentiation of ADSCs. The modified protocol improved differentiation efficiency and reduced the time required for differentiation of myocytes. It might be helpful to save cost and time when preparing myocytes for cell therapies and drug discovery.

## 1. Introduction

Muscle loss diseases such as sarcopenia, cachexia and atrophy are increased in the aging population and those with chronic diseases [1,2,3], and represent a serious clinical problem with few solutions. The homeostasis of skeletal muscle fibers is maintained by the continuous regeneration and activation of satellite cells, which are the muscle-specific stem cells that differentiate into myoblasts and form myotubes to replace the damaged myofibers [4]. Thus, the transplantation of skeletal muscle stem cells or progenitor cells is a potential therapy for muscular dystrophies [5,6]. Previous studies have reported the transplantation of muscle stem cell-derived myoblasts or myogenic cells in models of muscle injury [7,8]. Recently, mesenchymal stem cells (MSCs) have been suggested for use in cell therapies. These cells are derived from many organs, such as bone marrow, adipose tissue and umbilical cord blood and can differentiate into various lineages such as bone cells, cartilage cells, and fat cells [9] as well as muscle cells [10,11,12]. Many studies have reported that MSCs possess the ability to differentiate into the skeletal muscle cell lineage when treated with steroids such as hydrocortisone or dexamethasone [13,14,15]. Myogenic differentiation of MSCs is also promoted by co-culturing with skeletal myocytes, neonatal fibroblasts, or neonatal cardiomyocytes [16,17].

There are several animal models of muscular dystrophy, including non-mammalian (Caenorhabditis elegans, zebrafish, etc.) and mouse, dog, mouse and pig-based systems [18,19]. However, animal models cannot mimic human clinical, physiological, and biochemical manifestations [20]. Studies using animal models have inherently low throughput and are costly and time-consuming. Alternatively, in vitro cell models using fully defined biomimetic patient-derived cells are in the spotlight [21]. However, mature skeletal myocytes cannot be expanded in culture systems, and this limited property restricts the development of in vitro models for muscular dystrophies. Thus, differentiated stem cells are used for in vitro models of muscular dystrophy [22,23]. Myogenic differentiation using inducible pluripotent stem cells (iPSCs) for muscular dystrophy research and drug development have been widely used [24,25,26,27]. iPSCs are generated by introducing and expressing four specific genes, *Oct3/4*, *Sox2*, *Klf4*, and *c-myc* that cause reprogramming in somatic cells such as adult skin cells [28,29]. Because iPSCs are made through genetic manipulation, they have a problem with respect to safety of use, such as unpredictability and teratogenic potential in vivo [30].

MSCs, unlike iPSCs, are known to regulate immune responses, play a major role in the repair of damaged tissues, and have the potential to serve as useful tools in drug discovery [31]. Adipose tissue is a good source of MSCs [32,33,34], and adipose tissue-derived stromal/stem cells (ADSCs) are easier to obtain and isolate than MSCs derived from other tissues. ADSCs possess the capacity to differentiate into many lineages, including adipogenic, osteogenic, chondrogenic, myogenic, neurogenic and hepatogenic lineages [35,36,37]. To induce myogenic differentiation using ADSCs, a myogenic medium consisting of a mixture of 10% fetal bovine serum (FBS), 5% horse serum (HS) and 50 μM hydrocortisone in Dulbecco’s Modified Eagle Medium (DMEM), is commonly used [13,38]. However, a 6-week long period is required for the induction of differentiation. Thus, it would be advantageous to develop an alternative method that would shorten the period of differentiation.

Skeletal muscle secretes a number of cytokines (myokines) such as interleukin (IL)-1β, IL-6, IL-8, IL-10, and IL-15 [39]. Release of IL-6 is increased from skeletal muscle after prolonged exercise [40] and is known to be associated with stimulation of hypertrophic muscle growth and myogenesis of muscle stem cells [41]. Paradoxically, harmful effects of high doses of IL-6 have also been proposed, such as increased muscle wasting and atrophy [40]. In this study, we hypothesized that addition of IL-6 and/or myocyte-conditioned media may improve the myogenic differentiation efficiency of ADSCs in conventional medium.

## 2. Results

### 2.1. Combination of IL-6 and C2C12 CM Promoted Myogenic Differentiation

**Figure 1:**
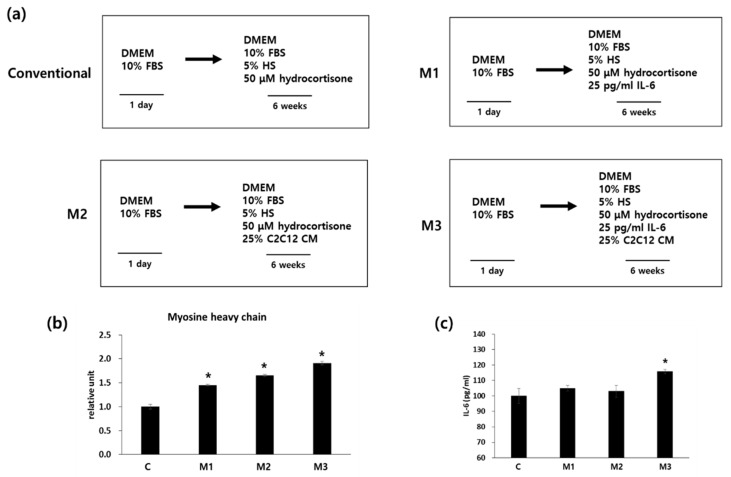
hADSCs were seeded and myogenic differentiation was induced for 6 weeks. (**a**) Schematic diagram of the differentiation protocols used. Protocol C is the conventional or common protocol. Protocols M1–3 are modified as indicated. (**b**) mRNA expression of myosin heavy chain in hADSCs after 6 weeks of differentiation. *n* = 3 independent preparations, each assay performed in duplicate. All values are expressed relative to the gene expression observed using by protocol C (1 unit). All treatments are significantly different from each other (**c**) After 6 weeks of differentiation, cells were incubated with serum-free DMEM for 24 h, the media were collected, and IL-6 levels were measured. * *p* < 0.05 vs. protocol C. DMEM: Dulbecco’s Modified Eagle Medium; HS: horse serum; FBS: fetal bovine serum.

There is evidence that IL-6 is involved in myoblast differentiation: *IL-6* gene expression is upregulated during C2C12 myoblast differentiation, exogenous IL-6 promotes myoblast differentiation, and inhibition of IL-6 mRNA expression by small interfering RNAs reduces C2C12 myoblast differentiation [42]. In addition, several studies have reported that the use of conditioned medium (CM) from myoblasts can induce myogenic differentiation of mesenchymal and embryonic stem cells. [43,44,45]. Therefore we examined whether the addition of IL-6 (protocol M1), C2C12 CM (protocol M2), or a combination of IL-6 and C2C12 CM (protocol M3) to the differentiation media would promote myogenic differentiation (Figure 1a).

After 6 weeks of differentiation, mRNA expression of myosin heavy chain (MYH), which is marker for myotubes, was checked by qRT-PCR analysis (Figure 1b). Addition of IL-6 (protocol M1) showed significantly increased expression of MYH compared with the conventional protocol (protocol C). Addition of C2C12 CM (protocol M2) significantly increased mRNA expression of MYH compared with protocols M1 or C. Addition of both IL-6 and C2C12 CM (protocol M3) showed the highest expression level of MYH compared with any of the other protocols. As IL-6 is a representative myokine [40], we measured the secretion of IL-6 as a myogenic differentiation marker. Addition of IL-6 (protocol M1) or addition of C2C12 CM (protocol M2) did not change the secreted IL-6 levels. However, the combination of IL-6 and C2C12 CM (protocol M3) significantly increased IL-6 secretion compared with the conventional differentiation medium (Figure 1c). These results suggest that addition of both IL-6 and C2C12 CM into conventional differentiation media improved myogenic differentiation of hADSCs.

### 2.2. Combination of IL-6 and C2C12 CM Reduced the Myogenic Differentiation Period

**Figure 2:**
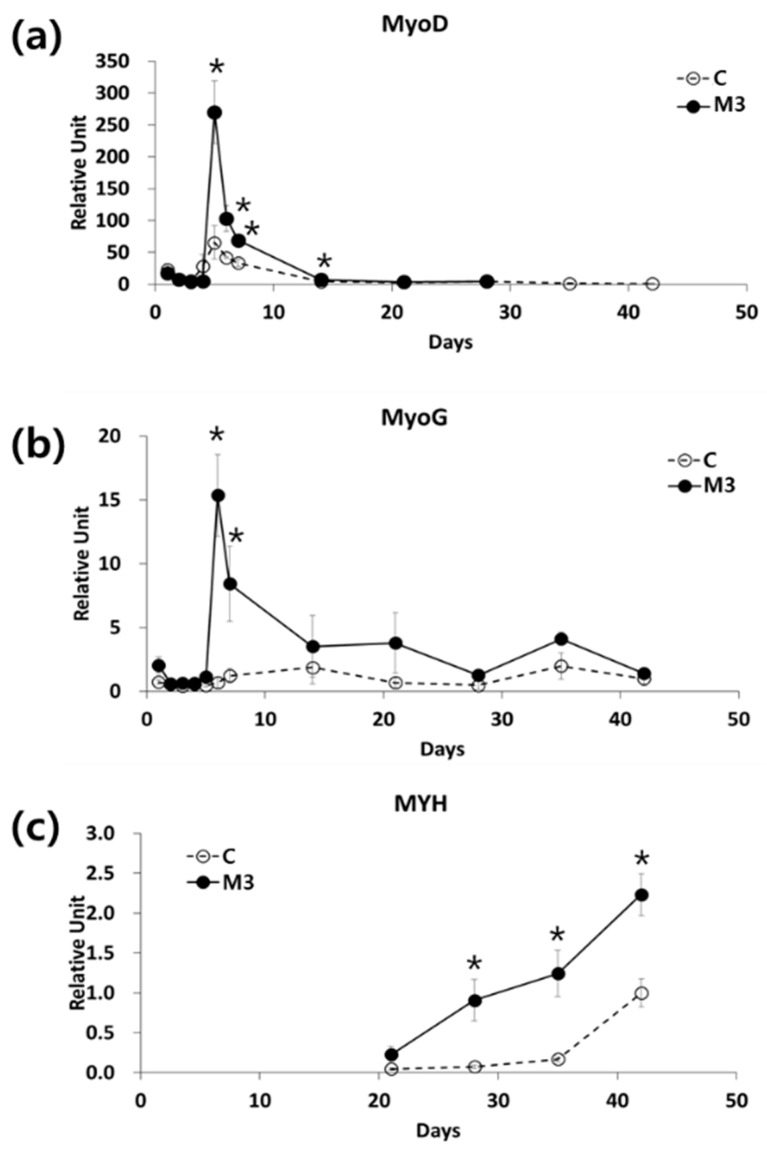
Effect of a combination of IL-6 and C2C12 CM on myogenic differentiation. mRNA expression of (**a**) MyoD, (**b**) myogenin (MyoG), and (**c**) myosin heavy chain (MYH) during myogenic differentiation, mRNA expression was analyzed by qRT-PCR. *n* = 3 independent experiments, each assay was performed in duplicate All values are expressed relative to the gene expression observed at 6 weeks using protocol C. * *p* < 0.05 vs. protocol C.

We checked the mRNA expression of MyoD, which is a myoblast marker, and MyoG, which is a marker of multinuclear muscle cells, at various times during differentiation using two different protocols—the conventional protocol (C) and the modified protocol using a combination of IL-6 and C2C12 CM (M3). For both protocols, the expression of MyoD mRNA reached a peak at day 5 after the initiation of differentiation and then gradually decreased. However, MyoD mRNA expression at day 5 was 7.2-fold higher using the M3 protocol than using the conventional protocol (Figure 2a). With the M3 protocol, the expression of MyoG mRNA peaked at day 6 and was 22.3-fold higher than the conventional protocol at this time. With the conventional protocol, MyoG mRNA expression did not peak until day 15, a much later time point than the M3 protocol (Figure 2b). The mRNA expression of MYH, a marker for mature myocytes, started to increase from day 21 using the M3 protocol, and by day 28 had already reached a level similar to that of day 42 using the conventional protocol (Figure 2c).

**Figure 3:**
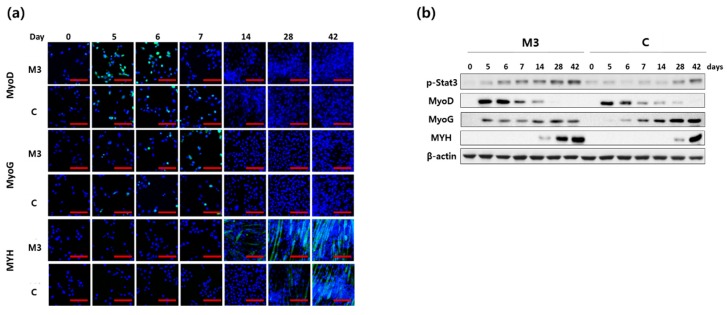
Effect of a combination of IL-6 and C2C12 CM on MyoD, MyoG and MYH protein expression during myogenic differentiation. (**a**) The expression of MyoD, MyoG and MYH were checked by immunofluorescence staining (scale bar, 200 μm) (**b**) The expression of MyoD, MyoG, MYH and p-STAT3 was analyzed by western blotting.

We next checked the expression of MyoD, MyoG and MYH protein expression by immunofluorescence staining (Figure 3a) and western blotting (Figure 3b). Similar to mRNA expression, MyoD and MYH protein expression was much higher using the M3 protocol. The expression of MYH protein at day 28 using the M3 protocol was similar to that of day 42 using the conventional protocol (Figure 3). MyoG protein expression started to increase earlier (at day 5) using the M3 protocol, but the expression level was lower than that using the conventional protocol on days 28 and 42 (Figure 3b). It is known that IL-6 may contribute to activation of the STAT3 signaling cascade and thereby myogenic differentiation [46]. The expression level of p-STAT3 protein, which is the activated form of STAT3, was found to be higher throughout the differentiation period in the M3 protocol compared to the conventional protocol (Figure 3).

**Figure 4:**
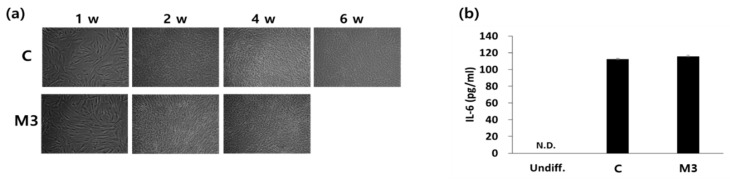
Effect of a combination of IL-6 and C2C12 CM on morphological changes during differentiation and on IL-6 secretion. (**a**) Morphological changes of cells during the differentiation process. (**b**) After 4 weeks (protocol M3) or 6 weeks (protocol C) of differentiation, media were changed to serum-free DMEM and cells were incubated for 24 h. Media were collected and IL-6 levels were measured. w: week(s).

In addition, myotube generation (Figure 4a) and IL-6 secretion levels (Figure 4b) were similar between the M3 protocol at 4 weeks of differentiation and the conventional protocol at 6 weeks of differentiation (Figure 4). These results indicate that the modified protocol using a combination of IL-6 and C2C12 CM enhanced myogenic differentiation and reduced the differentiation period.

### 2.3. Addition of STAT3 Activator Promoted Myogenic Differentiation

As we found that p-STAT3 expression was increased when we used the M3 protocol, it is possible that the STAT3 signaling pathway could be responsible for promoting myogenic differentiation Therefore, we used colivelin, a STAT3 activator, instead of IL-6 and C2C12 CM during myogenic differentiation of ADSCs.

**Figure 5:**
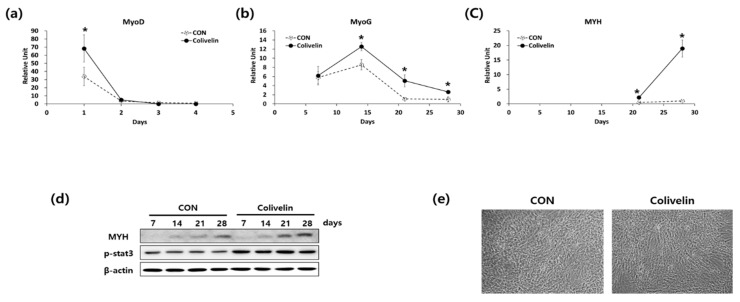
Effect of a colivelin on myogenic differentiation. (**a**–**c**) mRNA expression of (**a**) MyoD, (**b**) MyoG, and (**c**) MYH during myogenic differentiation. *n* = 3 independent experiments, each assay was performed in duplicate All values are expressed relative to the gene expression observed at 6 weeks using protocol C. * *p* < 0.05 vs. protocol C. (**d**) Cell morphology after 4 weeks of differentiation. (**e**) The expression of MYH and p-STAT3 was analyzed by western blotting.

Western blot analysis showed that addition of 50 nM colivelin increased p-STAT3 expression during the entire differentiation period compared to the conventional differentiation protocol (Figure 5d). The expression of mRNA levels of MyoD, MyoG, and MYH was also significantly increased during the differentiation period (Figure 5a–c). In accordance with the mRNA levels, the protein expression of MYH (Figure 5d) and myotube generation (Figure 5e) was also significantly increased by the addition of colivelin into the differentiation media. These results indicate that activation of STAT3 by adding colivelin to the differentiation medium can promote the myogenic differentiation of ADSCs.

## 3. Discussion

Because of advantages such as a large cell population and easy isolation, ADSCs have attracted attention for use in stem cell study. However, the long time period required for myogenic differentiation is one of the reasons why actual application for cell therapy or drug discovery for diseases involving muscle loss is difficult. In this study, we found that addition of IL-6 and myocyte-CM enhanced the myogenic differentiation of ADSCs through STAT3 activation. Similarly, we found that a STAT3 activator also promoted myogenic differentiation.

The exposure of mesenchymal stem cells to a myogenic environment such as co-culture with skeletal myoblasts is beneficial for myogenic differentiation [43,45]. Although the mechanisms are not yet fully understood, paracrine secreted cytokines and/or the extracellular matrix might affect the differentiation [11]. Muscle extracellular matrix scaffolds can recruit stem cells and induce differentiation, and this extracellular matrix scaffold promotes regeneration of other tissues such as bone cells depending on the surrounding environment [47].

In this study, the CM from C2C12 cells was beneficial to myogenic differentiation of hADSCs. In our experiment, the expression of MYH, the final marker of myogenic differentiation, was significantly increased by adding C2C12 myoblast CM to the conventional differentiation medium (Protocol M2). Analysis of the myoblast secretome or myotube matrix is needed in future studies to find a defined composition for promoting myogenic differentiation. It was reported that myogenic differentiation of ADSCs is induced by co-culture with primary myoblasts [43]. It is also well known that myokine, a cytokine produced and released by muscle cells, regulates muscle growth and regeneration [48]. Thus, other factors besides IL-6 in CM conditioning media are also likely to promote myogenesis, although IL-6 may play a major role.

Among the many secretory factors of myoblasts or myotubes, IL-6 is known to have two distinct functions on skeletal muscle. IL-6 promotes muscle satellite cell proliferation via regulation of cell-cycle-associated genes such as cyclin D1 and c-myc and stimulates muscle growth through IL-4, which promotes myoblast fusion [41,49]. Myoblasts from *IL-6* null mice show reduced differentiation and fusion capacities in vitro [46]. However, it is also known that an increase of circulating IL-6 concentrations or administration of high doses or long-term exposure to recombinant IL-6 leads to body weight loss and muscle atrophy [50,51]. Therefore, the role of IL-6 in muscle function and differentiation might be determined by its peripheral location and concentration. Thus, we hypothesized that a low dose of IL-6, similar to that secreted by autocrine in myoblasts or myotubes, could promote myogenic differentiation of ADSCs. The expression of MYH, the final differentiation marker of myogenic differentiation, was significantly increased by addition of low-dose IL-6 to the conventional differentiation medium (protocol M1) compared to the conventional protocol. The addition of IL-6 and C2C12 myoblast CM to the conventional differentiation medium resulted in better myogenic differentiation than when added alone, and 4 weeks of induction of differentiation was similar to that of induction of differentiation for 6 weeks using conventional differentiation medium.

Both undifferentiated and differentiated embryonic or mesenchymal stem cells have been tried for transplantation therapy for muscle dystrophy in both animal and human models and show a therapeutic effect [52,53,54]. Stem cells are generally differentiated to myoblasts, which are validated by the expression of MyoD, a myoblast marker [52,55]. Overexpression of MyoD itself in stem cells induces myogenic differentiation [56,57]. In this study, we found that MyoD is highly expressed 5 days after induction of differentiation when we added IL-6 and C2C12 CM, and the expression was about 7.2-fold higher than using the conventional differentiation protocol. Therefore, we speculate that transplantation of cells differentiated by the modified protocol might have better therapeutic effects. In the M3 protocol, MyoG protein expression appeared earlier, but the expression level was lower than that using conventional protocol from day 14. Recent reports suggest that MyoG-mutated cells have been successfully differentiated into terminally differentiated myofibers [58]. Therefore, we speculate that lower expression of MyoG during the M3 protocol may not significantly affect the terminal differentiation into myotubes. In addition, similar levels of expression of MYH, a myotube marker, were observed at 4 weeks of differentiation using the IL-6/C2C12 CM protocol, whereas 6 weeks were required using the conventional protocol, suggesting that the modified protocol exhibits the same differentiation efficiency over a shorter time period.

We found that the level of p-STAT3 protein increased during the entire differentiation period when IL-6 and C2C12 CM was used. The major downstream signaling pathway of IL-6 is the JAK/STAT3 pathway [40], and this signaling pathway is involved in muscle growth and differentiation as well as muscle atrophy and apoptosis [40]. Thus, it is thought that detailed regulation of the JAK/STAT3 pathway might be important to myogenic differentiation and function, and activation of this signaling pathway may improve myogenic differentiation. Colivelin is a neuroprotective peptide and activator of STAT3 [59,60]. When colivelin was added instead of IL-6 and C2C12 CM, the myogenic differentiation of ADSCs was improved over the conventional differentiation protocol. These results suggest that activation of STAT3 plays an important role in myogenic differentiation of ADSCs.

In summary, we found that a combination of IL-6 and C2C12 CM showed improvement of differentiation capacity compared with the conventional protocol, evidenced by the increased expression of MYH, myotube formation, and IL-6 secretion by STAT3 activation. As well, the STAT3 activator, colivelin, was able to induce the stimulation of myogenic differentiation of ADSCs. This modified protocol for myogenic differentiation might be helpful to save cost and time when preparing myocytes for cell therapies and drug discovery for skeletal muscle dystrophy. In addition, this protocol in combination with muscle extracellular matrix scaffold [6,47] for in vivo regeneration therapy for muscle dystrophy diseases and computer-aided design technology for in vitro research on biomedical scaffolds [61] would improve our techniques.

## 4. Materials and Methods

### 4.1. Differentiation and Tissue Culture Reagents

Hydrocortisone was purchased from Sigma (St. Louis, MO, USA). HS was purchased from Life Technologies (Grand Island, NY, USA). Phosphate-buffered saline, 0.25% trypsin/1 mM ethylenediaminetetraacetic acid (Trypsin-EDTA), DMEM, antibiotic/antimycotic solution and FBS were purchased from WelGENE (Daegu, Korea).

### 4.2. Myogenic Differentiation of hADSCs

The conventional myogenic differentiation protocol (protocol C) is as follows [13,38]. hADSCs, obtained from Invitrogen (Carlsbad, CA, USA), at passage 3–6 were seeded in 6-well plates (0.5 × 10^3^/cm^2^) and incubated overnight for cell adherence. The cells were then incubated in 10% FBS in DMEM for 1 day and then switched to the differentiation medium (10% FBS, 5% HS, and 50 μM hydrocortisone in DMEM). The differentiation medium was replaced every 2–3 days for 6 weeks. Protocol M1 was the same as the conventional protocol, except that IL-6 (25 pg/mL) was added to the differentiation medium and the medium was changed every 2–3 days for 5 weeks. For protocol M2, cells were incubated in 10% FBS in DMEM for 1 day and then switched to differentiation medium containing 10% FBS, 5% HS, 50 μM hydrocortisone in DMEM, and 25% C2C12 *v*/*v* cell-conditioned medium (C2C12 CM). The medium was replaced every 2–3 days for 6 weeks. Protocol M3 was the same as M2, except that IL-6 (25 pg/mL) was also added to the differentiation media. The differentiation medium was changed every 2–3 days for 5 weeks (Figure 1a). For STAT3 activation, 50 nM of colivelin (Tocris, Minneapolis, MN, USA) was applied to the existing differentiation media. The differentiation medium was changed every 2–3 days for 4 weeks

To produce C2C12 CM, mouse myoblasts (C2C12 cell line) were obtained from the American Type Culture Collection (ATCC, Rockville, MD, USA). Cells were grown at 37 °C and 5% CO_2_ in a humidified chamber in growth medium (DMEM supplemented with 10% FBS and 1% penicillin-streptomycin solution). Myogenic differentiation was induced on confluent cultured cells by changing the growth medium to differentiation medium (DMEM supplemented with 2% HS instead of FBS). The differentiation medium was replaced daily. C2C12 cells were differentiated for 1 week and then the culture media were collected 24 h after replacement and used for differentiation of hADSCs.

### 4.3. Measurement of IL-6 Levels

After 4 or 6 weeks of myogenic differentiation, the medium was replaced with serum-free DMEM. After 24 h of incubation, media were collected, and IL-6 levels were determined in duplicate using a human IL-6 ELISA kit (R&D Systems Inc., Minneapolis, MN, USA) according to the manufacturer’s instructions.

### 4.4. Quantitative Real-Time-PCR (qRT-PCR) Analysis

The total RNA was extracted from the cultured cells using TRIZOL reagent (Invitrogen) following the manufacturer’s instructions, and cDNA was synthesized using a PrimeScript 1st strand cDNA synthesis kit (Takara Bio Inc., Kyoto, Japan). qRT-PCR was performed using the SYBR Premix Ex Taq II, ROX plus (Takara Bio Inc.) and the Prism 7900HT sequence detection system (Applied Biosystems, Foster City, CA, USA). PCR was carried out for 40 cycles (2 min at 50 °C, 10 min at 95 °C, and 40 cycles of 10 s at 95 °C and 1 min at 60 °C). The relative copy number was calculated using the threshold crossing point (Ct) as calculated by ΔΔ*C*t. Primer sequences were as follows: 5′-GCCGCTAGAGGTGAAATTCTTG-3′ and 5′-CATTCTTGGCAAATGCTTTCG-3′ for human 18s ribosomal RNA; 5′-CCAGAGCTGAACCTTGAGGG-3′ and 5′- ACCTGCTACATTTGGGACCG-3′ for human MyoD; 5′-GATCATCTGCTCACGGCTGA-3′ and 5′-CCCGGCTTGGAAGACAATCT-3′ for human MyoG and 5′-TAAGGTCGCATCTCTACGCC-3′ and 5′-AAGGCTTGTTCTGGGCTTCA-3′ for human MYH.

### 4.5. Immunofluorescence Staining

The cells were fixed in 10% formalin. After antigen unmasking, the cells were permeabilized in 0.5% Triton X-100, and non-specific protein binding sites were saturated with protein block (Dako, Carpentaria, CA, USA) for 1 h. The cells were incubated with primary antibodies (1:100) overnight in a cold room, washed, and incubated with fluorescein isothiocyanate-conjugated secondary antibodies for 30 min. Antibodies were purchased from Santa Cruz Biotechnology (Santa Cruz, CA, USA). Nuclei were then fluorescently labeled with DAPI. The labeled cells were observed under a confocal microscope (LSM 700, Carl Zeiss Inc., Oberkochen, Germany).

### 4.6. Western Blotting

Cells were lysed with Mammalian Protein Extraction Buffer (GE Healthcare, Milwaukee, WI, USA) containing a protease and phosphatase inhibitor cocktail (Sigma-Aldrich, St. Louis, MO, USA). The total proteins (50 μg) were resolved by 6% sodium dodecyl sulfate polyacrylamide gel electrophoresis, transferred onto membranes, and blocked with 5% skimmed milk in Tris buffered saline containing 0.1% Tween-20. The membranes were incubated with specific primary antibodies and horseradish peroxidase-conjugated secondary antibodies. Antibodies were purchased from Santa Cruz Biotechnology (Santa Cruz, CA, USA). The membranes visualized by incubating with Immobilon Western Chemiluminescent HRP Substrate (Millipore, St. Charles, MO, USA). Chemiluminescence was detected by LAS-4000 (Fuji Film, Tokyo, Japan). The images derived from western blotting were analyzed through ImageJ software (National Institutes of Health, Bethesda, MD, USA) software for Windows.

### 4.7. Statistical Analyses

All data are expressed as mean ± standard error of at least three independent experiments. Data were analyzed using Analysis of Variance followed by post-hoc analysis using the Tukey range test (SPSS 10.0 statistical software). *p*-values less than 0.05 were considered statistically significant.

## Abbreviations

hADSCs	human adipose tissue-derived stem cells
FBS	fetal bovine serum
HS	horse serum
DMEM	Dulbecco’s Modified Eagle Medium
IL	interleukin
CM	conditioned medium
MyoG	myogenine
MYH	myosin heavy chain
MSC	mesenchymal stem cells
